# The impact of cognitive reserve in the recovery of chronic encephalopathy associated with traumatic brain injury – part one

**DOI:** 10.25122/jml-2022-1004

**Published:** 2022-04

**Authors:** Silvina Ilut, Irina Maria Vlad, Dafin Muresanu

**Affiliations:** 1. Department of Neuroscience, Iuliu Hatieganu University of Medicine and Pharmacy, Cluj-Napoca, Romania; 2. RoNeuro Institute for Neurological Research and Diagnostic, Cluj-Napoca, Romania

## Chronic Traumatic Encephalopathy (CTE)

### Definition

Chronic traumatic encephalopathy (CTE) represents a neurodegenerative disease that appears after repetitive head impacts. It is characterized by hyperphosphorylated tau (p-tau) deposits in the sulcus. Currently, CTE can be diagnosed based only on neuropathological examination.

The main categories of symptoms for CTE manifest as:

• Cognitive impairment at the mean age of 60, and then progressing to dementia;
• Behavioral changes at approximately the age of 35 and then progressing to cognitive impairment.

### Etiology

Recurrent closed traumatic brain injury (especially mild TBI) is the incriminated causative factor of CTE. Although, sometimes, the causative TBI can also be singular [1, 2]. 

Furthermore, CTE can be associated with post-traumatic encephalopathy or a single disease.

**Figure 1.**
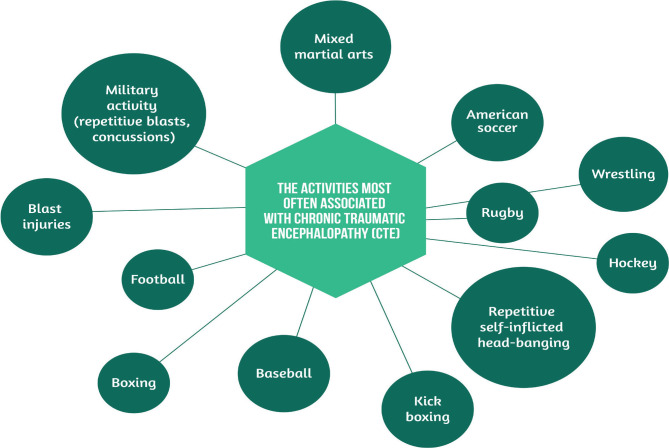
The activities most often associated with CTE.

Generally, the most incriminated activities associated with CTE are contact sports, but not exclusively (Figure 1) [3–6]. Other situations that correlate with CTE are the explosions encountered among military personnel and domestic abuse.

### Risk Factors

• Genetic risk factors (ApoE3 or ApoE4 allele);
• Age at the time of TBI (or repetitive TBI): younger or older age;
• Military service;
• Epilepsy;
• Length of sports participation;
• Head trauma [3].

### Pathophysiology & Epidemiology

From an epidemiological perspective, most cases of CTE among athletes, with a percentage of 30% [6], have microscopic lesions that comprise mechanical damage to neurons, glia cells, and brain blood vessels secondary to the applied forces (acceleration, deceleration, rotation).

Generally, from a pathophysiology point of view, the microscopic lesions leading to CTE comprise mechanical damage to neurons, glia cells, and brain blood vessels that are secondary to the applied forces (acceleration, deceleration, rotation). 

The distinct types of lesions, similarly to Alzheimer's disease, range from:

• Micro-bleeds;
• Axonal injury;
• Loss of blood-brain barrier;
• Inflammation;
• The accumulation of hyperphosphorylated tau (p-tau) protein;
• Neurofibrillary tangles.

Followingly, it is also called tauopathy.

Furthermore, the amount of traumatic brain injury that leads to CTE and other potential factors that might be causative agents is currently unknown [2–4].

### Clinical Presentation

The clinical manifestation of CTE varies from the most common cognitive and behavioral changes to disorders of sensation and motility.

The onset can overlap with signs and symptoms of the acute phase of traumatic brain injury, but it can also develop with a delay of up to tens of years after the episode of trauma. Usually, the onset is after about 8–10 years [7] and its age varies between the 20s and 70s, though most athletes already have the first signs and symptoms by the time they retire from sports practice [8].

**Figure 2.**
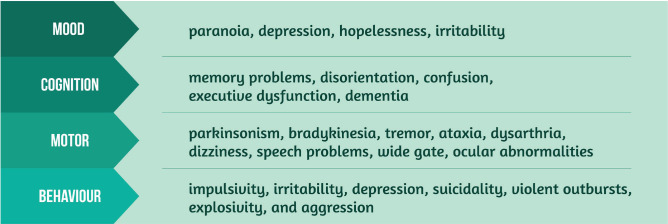
Clinical signs and symptoms of CTE.

Clinical signs and symptoms of CTE may include (Figure 2) [2, 5, 8, 9]:

• Mood;
• Cognition;
• Motor;
• Behavior.

### Diagnosis

Unfortunately, the diagnosis can currently be made only neuropathologically during the postmortem examination [3, 4, 10]. This is also one of the disadvantages regarding knowledge about this disease since the information is usually from retrospective cases and the statements of different family members of the affected patients. The imagery used in clinical practice does not bring information about the disease as it can seem to be normal.

### Prevention

The importance of CTE prevention stems from its critical consequences and its impact on the affected individuals and their families. In addition, the omission of this diagnosis during life in several patients also occurs.

The continuous progression of the disease is associated with significant changes in the quality of life ranging from:

• Social dysfunction;
• Financial problems;
• Paranoia;
• Suicidal ideations;
• Different phobias;
• Interpersonal relationship dysfunction (divorce, domestic abuse);
• Trouble with sleep;
• Substance misuse and abuse [8].

Several prevention strategies have been adopted, from the use of helmets and mouthguards to the establishment of different protocols for athletes that clear state rules that have to be followed before their reintroduction into play and to changes of rules in sports [11]. It is vital to have a multidisciplinary involvement, for example, in sports: from referees to coaches to policymakers and physicians. Additionally, because the military personnel are at risk during combat, physical exercises, other sports, or recreational activities, several protective gears and protocols are in place to ensure their protection as much as possible [12].

The second part will discuss the cognitive reserve and rehabilitation associated with CTE.
